# Curative Treatment of Phthisis Pulmonalis, and the Modus Operandi of Cod Liver Oil

**Published:** 1853-10

**Authors:** 


*Curative Treatment of Phthisis Pulmonalis, and the Modus Operandi of Cod Liver Oil.* By Prof. J. H. Bennett, (Am. Jour. Med. Sciences, from Monthly Jour. Med. Sciences.)

Prof. J. H. Bennett read an interesting paper on this subject before the Edinburgh Medico-Chirurgical Society, (Feb. 2, 1853,) in which he entered at considerable length into an exposition of those facts, which, in his opinion, proved the curability of tubercle. He considered its pathology to be, first, a derangement of nutrition, dependent on an excess of the albuminous, and a deficiency of the oily constituents of the chyle; secondly, an impaired constitution of the blood; so that, thirdly, when exudations occurred, they assumed the form of tubercle. He then stated that, in conformity with these views, he considered that the treatment of phthisis should be directed exclusively to the general system, and particularly to the correction of the impaired digestion and assimilation, while the local disease of the lung should, in most cases, be left to itself. The rule of practice, he thought, ought to be, an endeavor so to act on the digestive system as to improve the quality of the blood. This was to be accomplished, not only by giving animal oil, (which, however, as the principle of nutrition that was deficient, was directly indicated,) but by attention to those circumstances which correct acidity in the alimentary canal, and stimulate all nutritive processes, such as exercise, bathing, proper diet, mental occupations, &c. He considered the ordinary palliative medicines as useless, and even injurious, because they frequently interfered with the main object of the cure—the correction of the acidity in the intestinal canal, and the introduction into the system of the normal products of assimilation. Beef-steaks and mutton-chops were all very well so far as they went, but experience had proved that, in the majority of cases, they could not be digested. By giving cod-liver oil we saved the stomach, as it were, the trouble of extracting and reducing fluid fat from the food; and, although a considerable quantity of it was not assimilated, a sufficiency was absorbed to increase the molecular basis of the chyle, and to give a stimulus to the better elaboration of blood. It was with this view that he administered cod-liver oil; and since its introduction into British practice, he had had the satisfaction to see it extensively adopted by practitioners in this country, some of whom, as, for instance, Dr. C. J. B. Williams, of London, had published theories as to the action of this remedy, identical, in all essential particulars, with the one advanced by him, (Dr. B.) He then criticised at some length the remedies in common use, and pointed out that such as were beneficial operated by stimulating the nutritive functions. The good effects of change of climate, he thought, were to be entirely explained in this way, and not to any supposed action on the lungs. He concluded by announcing his intention of shortly laying his views and the result of his experience more at length before the profession, in a separate publication. —*Charleston Med. Jour.*

